# Supplementary material for: MetaRibo-Seq measures translation in microbiomes
Source: Nat Commun. 2020 Jun 29;11:3268. doi: 10.1038/s41467-020-17081-z (PMC7324362; doi:10.1038/s41467-020-17081-z)
Supplement: Supplementary file 10 — Supplementary Data 7 [file 41467_2020_17081_MOESM10_ESM.zip › File2/Confidence_High_Taxonomy/2364_out.krona.html]

Javascript must be enabled to view this page.

members
magnitude
magnitudeUnassigned
count
unassigned
taxon
rank

2364\_out

13

superkingdom
2
13

13
1239
phylum

186801
class
13

13
order
186802

family
31979
3

genus
1485
3

species
1262790

SRS019068\_contig\_number\_4932
1

2

SRS016335\_contig\_number\_contig-100\_18020.135133SRS148159\_contig\_number\_15901
1896984
species

1
186803

SRS013476\_contig\_number\_contig-100\_26506.26506
family
10

2

SRS024132\_contig\_number\_27220SRS147139\_contig\_number\_18075
species
1898203

7
genus
1
841

SRS077641\_contig\_number\_10249


SRS049959\_contig\_number\_50324SRS144537\_contig\_number\_40424SRS147766\_contig\_number\_16194
1262946
species
3

2
species
2293138

SRS143466\_contig\_number\_26468SRS147271\_contig\_number\_25501


SRS015431\_contig\_number\_17699
species
166486
1
